# A Pilot Study of an Attachment-Based Parenting Intervention for Mandarin-Speaking Families in Canada

**DOI:** 10.1177/13591045251400385

**Published:** 2025-11-24

**Authors:** Helen Liu, Lin Bao, Anna Kristen, Anh-Thu Vu, Marlene Moretti

**Affiliations:** 1 Department of Psychology, 1763 Simon Fraser University, Burnaby, BC, Canada

**Keywords:** adolescence, attachment, psychotherapy, parenting, immigrant

## Abstract

**Objectives:**

To address the gap in accessibility of mental health services for Mandarin-speaking families in Canada, this study evaluated the uptake, acceptability, and preliminary effectiveness of *Connect for Mandarin Speaking Families*, a culturally-inclusive and attachment-focused parenting program designed to promote youth and family wellbeing.

**Methods:**

Participants were 17 birth and 1 kinship immigrant parents from China to Canada (age 39–69, *M*_*age*_ = 49.42, *SD* = 7.25; 14 mothers; 4 fathers) seeking support for concerns about their children’s mental health problems (age 12–18, *M*_*age*_ = 14.44, *SD* = 1.80; 27.8% male, 72.2% female). Parents completed self-report pre- and post-measures assessing their child’s mental health and evaluated the perceived fit and helpfulness of the program in this single-arm pilot study.

**Results:**

Preliminary evidence was found for the program’s effectiveness in reducing adolescent emotional and behavioural problems and improving family satisfaction. Importantly, parent attendance and retention were high (85%), and parents reported the program was very helpful and a good fit with their cultural values.

**Conclusions:**

This pilot study is among the first to evaluate a culturally-inclusive, attachment-based parenting intervention for Chinese immigrant families in Canada, delivered in their native language. Future research with larger samples and further adaptations to address immigrant-specific family challenges is warranted.

## Plain Language Summary

**Why was the study done? **The study explored how Chinese immigrant parents with youth experiencing emotional and behavioural problems engaged in an attachment-based parenting program *(Connect) *that was delivered in Mandarin and adapted to align with Chinese cultural values and parenting norms. The researchers wanted to know if parents would attend and remain in the program, if they liked the program and found it helpful, and whether it improved their children’s behavioural and emotional problems and family functioning. **What did the researchers do?** The researchers recruited 18 participants (17 birth parents and 1 kinship parent, between 39 to 69 years old) from three *Connect for Mandarin-Speaking Families *groups that ran in Canada. These parents were seeking support for concerns related to their children’s mental health, with the children ranging in age from 12 to 18 years old. Participants were given questionnaires to fill out before and after they participated in the 10-week parenting program. **What did the researchers find? **Results from attendance sheets and parent feedback showed that parents attended consistently and found the program to be helpful. Most parents believed that the program aligned with their culture. Results from the questionnaires showed that there were decreases in teen’s emotional and behavioural problems at the end of the program and increases in family satisfaction. Because the number of participants in the study was so small, we should be cautious in drawing conclusions from the results. **What do the findings mean? **This was a small, preliminary study, but it suggests that the culturally tailored version of *Connect* may be a promising avenue for supporting Mandarin-speaking families in Canada. The researchers recommend further studies with a larger number of participants so that the results can be interpreted with more confidence.

## Introduction

In our increasingly globalized world, immigration experiences can often involve a multitude of challenges for parents and children. Nearly 25% of the Canadian population are immigrants (Government of Canada SC, 2022), and recent estimates show up to 16% of immigrant adolescents (first- and second-generation) struggle with diagnosable mental health disorders (Gadermann et al., 2022). Chinese Canadians comprise 4.6% of the population (Government of Canada SC, 2022), pointing to the need for culturally sensitive and accessible parenting programs.

Adolescence is a vulnerable period for the onset of mental illness (Solmi et al., 2021), and acculturative stress associated with ongoing demands of bicultural environments and identities may increase the risk for mental health challenges amongst some first- and second-generation immigrant youth (Lerias et al., 2024). While there is mixed evidence on the impact of parent-child acculturation gaps on child and youth functioning (Shukla et al., 2025), when immigrant children and their parents struggle, many barriers hamper their access to mental health services. Stigma and discrimination have been recognized as barriers to accessing mental health services in China, and government initiatives to increase mental health literacy are underway (Zhang et al., 2025). Not only might these concerns linger among immigrant Canadians, but other barriers including lack of knowledge about available services, difficulties navigating unfamiliar service delivery systems, costs, and language accessibility may contribute to their underutilization of mental health services (Yang et al., 2020; Zhang et al., 2025).

Parenting support that is culturally inclusive and delivered in the native language of Chinese immigrants may mitigate barriers and increase the accessibility of effective programs. A growing body of literature demonstrates the effectiveness of attachment-based parenting programs (ABPPs) in promoting attachment security and improved mental health amongst children (Jugovac et al., 2022). Importantly, ABPPs have been demonstrated to promote transdiagnostic improvements across multiple domains of parent and youth mental health and wellbeing, and thus may align with economically viable health promotion strategies.

As attachment theory and its applications have evolved, the appreciation of culture, its historical contexts, and its impacts on parenting, have deepened. While the evolutionary importance of attachment is universally acknowledged, concerns about the cross-cultural generalizability of basic tenets of attachment theory (e.g., Keller, 2018; Otto & Keller, 2014) have been fruitful in promoting greater recognition of the importance of attachment with multiple caregivers – as opposed to the ‘primary parent-child dyad’ – in determining child wellbeing. Criticisms of attachment theory have also pushed the field to recognize differences between individualistic versus collective cultures in their valuing of independence versus interdependence as parenting goals and indicators of healthy child development (Quinn & Mageo, 2013; Wu & Chao, 2017). Some theorists have also raised questions about the applicability of cornerstone concepts including caregiver sensitivity. However, recent models suggest that overall differences between countries and cultural orientations in parenting do not necessarily negate the importance of individual caregiving qualities in promoting secure attachment (Mesman et al., 2016, 2018). For example, even though Chinese parents may, on average, use psychological control and authority assertion more frequently than parents from Western cultures (Ng et al., 2013), caregiver warmth and sensitivity within families likely exert similar effects across cultures. Consistent with this view, Waters et al. (2025) confirmed that amongst middle-class Chinese families, individual differences in maternal sensitivity with infants assessed at 14–24 months predicted individual differences in security of children’s attachment representations at age ten. Nonetheless, the importance of capturing cultural nuances in caregiving is critical in the development of culturally inclusive programs that support parents through a shared understanding of cultural belief systems and caregiving goals and practices.

*Connect* is an evidence-supported, manualized, attachment-based intervention for parents and other caregivers of children and youth experiencing significant behavioural and emotional problems (Barone et al., 2020, 2021; Moretti et al., 2015; Pasalich et al., 2021). Delivered by two trained facilitators in a small group-based format, the program includes a welcome meeting, nine 90-min therapeutic sessions, and a final feedback session. The program targets the building blocks of attachment security, including parent reflective functioning, sensitivity, dyadic affect regulation, and parent-teen mutuality and partnership. Each session introduces a principle related to attachment and adolescent development, and includes emotionally evocative role-plays, reflection exercises, and group discussions to build parents’ awareness of adolescent attachment needs and promote collaborative problem-solving. Importantly, while strengthening the parenting building blocks that promote attachment security, *Connect* does not adopt a psychoeducational prescriptive approach. Rather, it adopts an emotionally focused stance that models and strengthens reflective function to enable parents to “step into” their child’s state of mind, providing opportunities for parents to practice sensitivity and responsiveness to children’s attachment needs, while regulating challenging emotions that may arise in themselves or their child. The program can be delivered in-person or online (*eConnect*; Bao & Moretti, 2023), with the online version retaining all program components.

A series of uncontrolled, waitlist, and randomized clinical studies have provided strong evidence for the effectiveness and efficacy of *Connect* (Level 1–Supported Program; California Evidence-Based Clearinghouse for Child Welfare, 2022) in reducing youth externalizing and internalizing problems, attachment insecurity, parent and youth affect dysregulation, and parent depressed mood and stress, with retention of treatment gains up to two years post-treatment (Bao & Moretti, 2023; Högström et al., 2017; Moretti et al., 2015; Pasalich et al., 2021). Building on this evidence, surface modifications of *Connect* have been undertaken across a variety of countries and cultures to ensure cultural inclusiveness and responsiveness while retaining deep program structure and integrity. To guide these adaptations, the Cultural Adaptation Framework proposed by Barrera et al. (2013) was employed. This framework emphasizes an iterative process that prioritizes both fidelity and cultural fit, and outlines five key steps: (1) information gathering and community input; (2) preliminary adaptation design; (3) testing the preliminary adaptation with a pilot group; (4) refinement based on feedback and outcomes; and (5) cultural adaptation trial with continued refinement. Guided by this five-step approach, modifications to *Connect* in each country were developed through collaborative partnerships with cultural knowledge holders, practitioners, local clinical scientists, and government stakeholders, and informed by parent feedback. Research has demonstrated comparable outcomes across diverse populations, including forcibly displaced caregivers in Sweden (Kristen et al., 2023; Osman et al., 2017, 2021), kinship caregivers in Australia (Pasalich et al., 2021), and birth parents in Italy and Sweden (Barone et al., 2021; Benzi et al., 2023; Ozturk et al., 2019). Preliminary evaluations of culturally adapted versions of *Connect* have also shown promise in Mexico and South Africa (Gallegos Guajardo et al., 2023; Haffejee et al., 2024).

The current study builds on prior research with clinical researchers and practitioners in Beijing, China (Bao et al., 2025) that included the translation of the *Connect* program into Mandarin and in-depth tailoring to align with and ensure representation and respect for Chinese cultural beliefs pertinent to parenting children, all while retaining deep program structure. The modifications emphasized clarity and resonance by using more specific language and weaving in culturally grounded examples, idioms, and poems to illustrate abstract concepts. Sessions placed greater attention on empathy and emotional reflection, more explicitly prompting parents to consider emotions rather than problem-solving. Role-plays were modified to align with cultural norms, and based on parent feedback, suggestions for at-home practice were also provided. Results from pilot groups in Beijing demonstrated high program uptake, acceptance, and perceived cultural fit through indicators such as attendance, retention, and parents’ feedback. Parents also reported significant decreases in overall youth internalizing (*g*_*a*_ = 0.43) and externalizing problems (*g*_*av*_ = 0.60).

Given these promising findings, the current research aimed to evaluate the cultural fit, uptake, and acceptability of *Connect for Mandarin-Speaking Families* amongst Chinese immigrant families in Canada seeking services in relation to their child’s mental health. The study also explored preliminary program impact on youth mental health problems and family functioning.

## Methods

The current study is a part of a larger international research program evaluating the impact of the online version of *Connect* on youth mental health (Bao & Moretti, 2023; Benzi et al., 2023; Kristen et al., 2023).

### Recruitment

Mandarin-speaking parents of youth aged 8-18 years in Canada who were seeking services for concerns about their child’s mental health problems were recruited to enrol in *Connect for Mandarin Speaking Parents.* Once parents were enrolled in the group, they were informed of the opportunity to voluntarily participate in the research study; inclusion in the program was not subject to parent participation in the research. Exclusion criteria included low child intellectual functioning (IQ <70), major mental disorder (schizophrenia, bipolar disorder), or acute psychosis.

### Procedures

Parents who expressed interest in research participation completed written informed consent. Parents completed an online questionnaire package of program outcome measures assessing youth mental health and family functioning prior to starting the group (T1) and after completing the group (T2). In addition, after completing the group, parents completed feedback questionnaires related to the perceived fit and helpfulness of the program. All parents received a $20 CAD honorarium for completing research materials at each of the two time-points. All research protocols and procedures received approval from the Simon Fraser University Office of Research Ethics [#2011s0284 and #20200401].

### Participants

In total, 33 parents enrolled across three *eConnect* groups that were delivered between February 2022 to April 2023. Twenty-three of these parents consented to participate in the research study, however, five of them failed to complete any or all of the pre-program questionnaires and were excluded from the quantitative analyses. The remaining 18 parents completed pre- and post-program questionnaires, and program feedback questionnaires were completed by 17 of these parents. Demographic data is only available for parents who participated in the research.

The final sample included 17 biological parents and 1 adoptive kinship parent (aged 39–69) of youth aged 12–18. All parents were born in China. Immigration duration ranged from 6 months to 40+ years. Demographic information is outlined in Table 1.

**Table 1.** Pre-intervention Demographic Characteristics of Sample

Variables	
Parents, n (%)	
Mothers	14 (77.8)
Fathers	4 (22.2)
Participant age in years (mean ± SD)	49.28 ± 7.42
Years spent in host country (mean ± SD)	11.25 ± 9.89
Highest education level, n (%)	
Partial elementary education	1 (5.6)
Partial completion of college/university	3 (16.7)
Completion of a college diploma or bachelor’s degree	12 (66.7)
Post-graduate degree completion	2 (11.1)
Financial well- being, n (%)	
Not enough money to cover living expenses	2 (11.1)
Barely enough money to cover living expenses	2 (11.1)
Enough money to cover living expenses	13 (72.2)
Not reported	1 (5.6)
Multiple caregiver homes, n (%)	6 (33.3)
No. of children in house (mean ± SD)	1.68 ± 0.80
Child sex: female, n (%)	13 (72.2)
Child age (mean ± SD)	14.4 ± 1.80
Child living in the parents’ home, n (%)	17 (94.4)
Child attending regular school, n (%)	14 (77.8)
Place of birth: outside Canada, n (%)	15 (83.3)
Years spent in host country (mean ± SD)	7.00 ± 3.79

### Intervention Modifications & Delivery

As previously noted, the initial translation and modification of the program was completed in collaboration with mental health professionals in Beijing who guided each step of the process with their cultural expertise. They also relied on parent feedback to inform iterative revisions to enhance cultural relevance while preserving the program’s structure and core therapeutic components (e.g., role plays, reflective exercises). Modifications included greater specificity in language and the use of cultural examples, idioms, and poems to elucidate key program ideas and concepts. For example, the poem “不识庐山真面目, 只缘身在此山中” (i.e., of Mountain Lu we cannot make out the true face, for we are lost in the heart of the very place) was used to illustrate the idea that stepping back from one’s immediate emotional reactions can create the space needed to better understand the perspectives of their teenagers. Another key adaptation was greater emphasis on using empathy instead of problem-solving. For instance, reflection questions used the word “情绪” (i.e., emotion/mood) rather than “感受” (i.e., feeling), and explicit prompts were embedded into the program to encourage parental reflection on emotions (e.g., the question “When we see a conflict coming, what can we do to respond to strengthen our relationship?” was revised to “When you and your child are about to get into a conflict situation, what kind of communication method can maintain or strengthen your relationship, rather than hurting it? What could you do? What body language, facial expression, and tone of voice might you use?”). Lastly, role-play scenarios were modified to ensure consistency with cultural parenting norms and practices (e.g., conflict over schoolwork instead of conflict over quitting a soccer team).

*Connect for Mandarin-Speaking Parents* groups were delivered by mental health practitioners from hospitals, community mental health agencies, and school counselling services, all of whom completed a standardized 20 hour *Connect* training workshop. Trainees also received session-by-session weekly supervision for 1 hour based on their supervisor’s review of videotaped sessions to ensure program adherence and intervention quality. Supervision was provided by the same clinician across all three *Connect for Mandarin-Speaking Parents* groups delivered in the study, and all trainees successfully achieved certification. All groups were delivered online via Zoom.

### Measures

All evaluation measures were used across previous studies evaluating *Connect* program outcomes, translated and back-translated by author L.B. in collaboration with the team of trained *Connect* facilitators in Beijing.

#### Attendance and Retention

Uptake of the intervention was assessed by program attendance and retention. Program retention was defined by parent attendance at least 6 of the 9 therapeutic sessions.

#### The Brief Child and Family Phone Interview (BCFPI)

The BCFPI (Cunningham et al., 2009) is a 36-item standardized parent-report measure assessing youth’s internalizing (T1: *α* = .91, T2: *α* = .91) and externalizing problems (T1: *α* = .92, T2: *α* = .92). In the current study, the six subscales (internalizing subscales: separation anxiety disorder, general anxiety disorder, major depressive disorder; externalizing subscales: attention deficit/hyperactivity disorder, oppositional defiant disorder, conduct disorder) showed good internal consistency (T1: *α* ≥ .76; T2: *α* ≥ .80). Parents rated the frequency of behaviours (at T1: over the past six months; at T2: over the past two weeks) on a 3-point scale (1 “Never; 3 “Often”).

#### Family Satisfaction Scale (FSS)

The FSS (Carver & Jones, 1992) is a unidimensional 10-item questionnaire that measures the satisfaction of family members across various aspects of family functioning such as flexibility, cohesion, and communication (“How satisfied are you, on average, with the following: e.g., The quality of communication between family members”; T1: *α* = .91, T2: *α* = .97). Items are rated on a 5-point scale (1 “Almost never”; 5 “Almost always”) for the past 6 months at T1, and the past two weeks at T2.

#### Parental Program Acceptability Questionnaire (PPAQ)

The PPAQ is 5-item self-report questionnaire assessing acceptability of the *Connect* program (Bao, 2023). We used the 3-item subscale previously identified via factor analysis to measure parents’ satisfaction with *Connect* (e.g., I am satisfied with *Connect*) rated on a 7-point scale (1 “Strongly disagree”; 7 “Strongly agree”).

#### Connect Treatment Engagement and Client Satisfaction Questionnaire

The Connect Treatment Engagement and Client Satisfaction Questionnaire is a 15-item self-report questionnaire (Moretti et al., 2020). On a 4-point scale (1 - “Very Helpful”; 4 - “Unhelpful”), parents rated the helpfulness in learning about attachment to enhance understanding of their teen and themselves as parents, and the helpfulness of various program components. Further, on an additional 4-point scale (1 – “A great deal”; 4 – “Not at all”), parents rated the extent to which they applied what they learned in their parenting, experienced or anticipated changes in the parent-teen relationship, and confidence in their parenting abilities increased. Parents also rated the degree to which they felt safe, welcomed, and respected in the group. Finally, parents responded to an open-ended question about perceived cultural fit: *“As a Chinese parent, how consistent is the content taught in the Connect parent group with your values, philosophy, and approach to raising children?”*.

### Statistical Analyses

Descriptive analyses were used for pre-intervention demographic information. Missing data across variables ranged from 5.6% to 22.2% and were found to be missing completely at random (MCAR) via Little’s MCAR test (chi-square (392) = .000, *p* = 1.000; Little, 1988). In line with leading recommendations (Little, 2013), 100 imputed datasets were generated and then pooled for analyses using multiple imputation (Manly & Wells, 2015). Paired sample *t*-tests were conducted to determine pre-post-program changes.

Effect sizes are reported using *Hedge’s g* statistic, with the following thresholds: *g* = 0.2 (small), *g* = 0.5 (medium), *g* = 0.8 (large; Hedges, 1981). The *Hedges’ g* statistic was selected over *Cohen’s d,* as the latter tends to provide biased (inflated) estimates with small samples (Cumming et al., 2012).

## Results

### Attendance and Retention

Out of 33 parents enrolled in the groups, 85% (*N* = 28) completed the program. On average, the parents attended 7 out of the 9 therapeutic sessions (*SD* = 1.85).

### Pre-to Post-program Change in Youth & Family Functioning

#### Changes in Youth Mental Health Problems

Parent reports of overall youth internalizing problems (see Table 2) decreased significantly from T1 to T2 with a large effect size (*p* < 0.001, *g* = 0.90). Parent reports of overall youth externalizing problems decreased significantly after the program with a medium to large effect size, *p* = 0.003, *g* = 0.78.

**Table 2.** Descriptive Statistics of Outcome Measures at T1 and T2 and Intervention Outcomes Using Paired Sample T-Test (N = 18)

Outcomes	Pre-intervention	Post-intervention	Paired sample t-test results			Effect size
	*Mean (SD)*	*Mean (SD)*	*S*_*mean*_	*SE*	*p*	*Hedge’s g*
BCFPI – INT	5.49 (1.13)	4.65 (0.85)	−0.84	0.21	<0.001	0.90
BCFPI – SAD	1.46 (0.44)	1.24 (0.30)	−0.22	0.11	0.053	0.47
BCFPI – GAD	2.15 (0.53)	1.87 (0.42)	−0.27	0.10	0.004	0.74
BCFPI – MDD	1.88 (0.47)	1.53 (0.32)	−0.44	0.13	0.004	0.75
BCFPI – EXT	5.77 (1.07)	5.10 (0.97)	−0.67	0.26	0.003	0.78
BCFPI – ADHD	2.21 (0.57)	2.01 (0.57)	−0.20	0.11	0.081	0.42
BCFPI – ODD	2.42 (0.45)	2.07 (0.49)	−0.35	0.10	0.003	0.79
BCFPI – CD	1.14 (0.21)	1.03 (0.08)	−0.11	0.05	0.031	0.53
FSS	24.05 (6.27)	28.45 (7.11)	−4.40	1.08	<0.001	0.92

*Note.* BCFPI: Brief Child and Family Phone Interview; INT: Youth internalizing problem subscale; SAD: Separation Anxiety subscale; GAD: Generalized Anxiety subscale; MDD: Depressive Mood subscale; EXT: Youth externalizing problem scale; ADHD: Attention Deficit Hyperactivity subscale; ODD: Oppositional Defiant subscale; CD: Conduct Problems subscale; FSS: Family Satisfaction Scale.

#### Changes in Family Satisfaction

Parents reports demonstrated a significant improvement of family satisfaction after the program, with a large effect size (*p* < 0.001, *g* = 0.92).

### Parent Ratings of Program Quality and Satisfaction

Seventeen parents completed feedback questionnaires related to the perceived fit and helpfulness of the program.

#### Program Acceptability

Most parents reported feeling satisfied with the program (strongly agree: 29%; agree: 59%; somewhat agree: 6%, and neutral: 6%). Most parents said the program met a previously unmet service need (88%), and that they would recommend the program to other families (94%).

#### Helpfulness of the Program Components

All parents (100%) found learning about attachment, and exploring its relevance to both their children’s and their own behaviors, helpful. Parents found all program components helpful, including roleplays (very helpful: 59%; helpful: 41%), reflection exercises (very helpful: 35%; helpful: 65%), flipcharts (very helpful: 47%; helpful: 53%), and handouts and at-home practice suggestions (very helpful: 29%; helpful: 71%). All parents reported the program was helpful in understanding their children (a great deal: 82%; somewhat: 18%) and themselves as parents (a great deal: 65%; somewhat: 35%). Additionally, all parents reported applying what they learned in the program when parenting (a great deal: 35%; somewhat: 64%), and all parents observed changes in their relationship with their children (a great deal: 24%; somewhat: 76%). Finally, all parents reported increased confidence in their parenting (a great deal: 53%; somewhat: 47%) and anticipated future improvements in their relationship with their children because of *Connect* (a great deal: 35%; somewhat: 65%).

#### Parent Sense of Safety and Respect

All parents (100%) agreed that their experience as a caregiver was respected in the group (a great deal: 82%; somewhat: 18%). Most parents (94%) felt comfortable and safe in the group to discuss their experiences and concerns.

#### Cultural Fit

Most parents (88%; *N* = 15) provided specific comments in response to the open-ended item about cultural fit, while some (12%; *N* = (2) did not provide any feedback. Most parents (80%; *N* = 12) reported that the program was a good fit with Chinese cultural values and parenting approaches. For example, one parent stated that the program “*deepens and expands on some of the concepts and skills I have learned about teaching and communicating with children before.”* Some parents (20%; *N* = (3) noted differences between their cultural background and the program content. However, despite these differences, two of the parents suggested that the program offered an approach that was aligned with their parenting goals. One parent stated *“It’s not the same. But after learning, I feel that the concept and approach of Connect is better.”* The other parent expressed, *“It is very different, but I think it is a healthier way that values equality, respect, and connection. Children like it very much, but it takes some time for us to change.”*

## Discussion

This pilot study examined the uptake, acceptability, and preliminary outcomes of a 10-week attachment-based parenting program originally adapted for Mandarin speaking families in China and delivered to Chinese immigrant parents in Canada. Our results show high program uptake with 85% of the enrolled parents completing the program, and on average, attending at least 7 of the 9 therapeutic sessions. Our results also indicate high program acceptability, with the majority of parents reporting that the program met their previously unmet service needs and almost all parents reporting that they would recommend the program to other families with similar needs. These findings highlight the program’s potential in increasing accessibility and mitigating barriers to access for Chinese immigrant families living in Canada. Previous research has shown that cultural and language barriers are common factors that limit immigrant families from accessing mental health services in Canada (Gyan et al., 2023), and incorporating culturally appropriate practices and offering services in their native language can increase the acceptability and effectiveness of the intervention among the target population (van Mourik et al., 2017).

In this study, all parents reported that learning about attachment enhanced their understanding of their children and themselves as parents. All program components were found to be helpful – especially roleplays, reflection activities, and take-home exercises – and parents reported applying what they learned to their own parenting. Parents reported increased parenting confidence, improved parent-child relationships, and hope for continued relational improvements. Further, the group environment was generally experienced as respectful and safe. Taken together, the present findings indicate that the program’s focus on attachment was resonant for parents and translated to meaningful changes in their confidence as parents and in their relationships with their youth, underscoring that broader implementation of the program may be a worthy avenue for further exploration.

The majority of parents reported that *Connect for Mandarin-Speaking Families* fit with their cultural values and approach to parenting. However, three parents felt the program’s approach was different from their traditional parenting philosophy. Interestingly, two of these three parents acknowledged that, despite the differences from their traditional parenting philosophy, the approaches and values promoted in *Connect –* such as equality, respect, and connection *–* were valuable, noting that changes in their parenting behaviors were well-received by their children, but that it would require time for the family to consolidate. Collectively, the pattern of parental responses suggests that the majority experienced the program as reaffirming their cultural values and parenting goals, and the comments of a few parents suggest that they may have discovered new ideas that they were integrating in their approach to parenting their child. These findings may reflect a process of cultural bridging, in which parents selectively integrate new relational strategies into their existing value systems (Phinney et al., 2000; Wu & Chao, 2011). These comments may also be reflective of the unique parenting experiences Chinese immigrants in North America encounter compared to their counterparts who remain in their home country – the population for whom *Connect for Mandarin-Speaking Families* was originally designed. Future studies might investigate whether more directly addressing immigrant-specific family challenges (e.g., acculturation conflicts, discrimination, social isolation) may enhance the program’s cultural and contextual relevance for immigrant families.

The study also investigated preliminary program impacts on youth mental health and family satisfaction, measured pre- and post-program. Due to the small sample size (*n* = 18), these results should be interpreted with some caution and future research is required to confirm and extend results. Our findings provide preliminary support for the effectiveness of *Connect for Mandarin-Speaking Families* in improving the mental health of youth of Chinese immigrant parents, with youth internalizing and externalizing problems significantly decreasing post-intervention with medium to large effects. The present findings are generally consistent with other studies evaluating *Connect*, including with forcibly displaced parents living in Sweden (Kristen et al., 2023; Osman et al., 2017) and parents in Beijing (Bao et al., 2025). It is notable that the effect size for reductions in internalizing problems is substantially larger in the current study (*g* = 0.90) than in the Sweden (*d* = 0.16) or Beijing (*g*_*av*_ = 0.43) studies. This is particularly important given research showing that when Asian immigrant youth develop mental health problems, they tend to exhibit higher rates of clinically impairing internalizing symptoms compared to immigrant youth from other ethnic backgrounds (Huang et al., 2012).

Finally, we found that family satisfaction significantly increased post-intervention (*g* = 0.92), reflecting greater satisfaction of various aspects of family functioning such as flexibility, cohesion, and communication. Similar findings were demonstrated in a pilot study delivering *Connect* for forcibly displaced families in Sweden (e.g., increases in open communication and emotional closeness; Kristen et al., 2023). For immigrant families navigating the stress of a child’s mental health challenges, access to a culturally grounded program may offer meaningful relief and contribute to increased satisfaction and mutual understanding within the family unit.

A notable strength of this study is intervention delivery in parents’ native language. In Canada, few mental health services and parenting interventions are offered in languages other than English (Canadian Mental Health Association, 2018), creating a barrier for immigrants who may feel unable or uncomfortable accessing services in their second language (Chen et al., 2009; Li & Browne, 2000). Discussing sensitive topics like mental health and familial conflict in one’s native tongue can reduce cognitive load and increase feelings of safety (George et al., 2015). Further, there is limited research on interventions in Canada that are designed to reflect the cultural values and parenting norms of Chinese families (Shih, 2018). Culturally adapted interventions can alleviate misunderstandings and reduce the fear of judgment that often prevents Chinese immigrant families from seeking help (Chen et al., 2009; Liu et al., 2020).

As the first effort to implement and evaluate the culturally tailored version of *Connect for Mandarin-Speaking Families* in Canada, this study had several limitations. First, the small sample and lack of comparison or control group necessitate caution in the interpretation of the findings. Second, our study only included parent-reported data. Future research would benefit from including multi-informant data, such as youth perspectives, to better understand program impacts and reduce the risk of reporting bias. Third, our sample included a wide range of immigration duration (6 months to 40+ years), which could have made it challenging to address the diverse experiences and needs in the group. Future research may wish to explore how immigration-related variables (e.g., recency and duration of settlement) influence perceived program fit or outcomes. Importantly, we did not include a parent-report measure of attachment, a key driver of change of the *Connect* program, despite previous studies in North American (Bao & Moretti, 2023; Moretti et al., 2015; Pasalich et al., 2022) and Italian samples (Barone et al., 2021; Benzi et al., 2023) consistently showing reductions in attachment anxiety and avoidance using the Adolescent Attachment Anxiety and Avoidance Inventory (AAAAI). The measure was not included due to concerns about its psychometric properties and sensitivity in detecting culturally meaningful changes in parent-reported youth attachment in our prior research in Beijing (Bao et al., 2025) and with Somali, Dari, and Arabic-speaking parents in Sweden (Kristen et al., 2024). Despite these limitations, the current study underscores the importance and value of offering culturally tailored services and represents an important step toward providing effective and accessible supports for immigrant families within Canada’s increasingly diverse society.

## Data Availability

Data are available upon reasonable request. The data are not publicly available due to privacy and ethical restrictions.*
